# Gellan Gum/Alginate Films Containing Biogenic *uva ursi* Silver Nanoparticles: Analytical Characterization and Antiviral Activity Against HSV-1

**DOI:** 10.3390/molecules31091459

**Published:** 2026-04-28

**Authors:** Roberta Della Marca, Francesco Busto, Carla Zannella, Stefano Liotino, Maria Chiara Sportelli, Muhammad Shoaib, Shahab Bashir, Massimiliano Galdiero, Elvira De Giglio, Anna De Filippis

**Affiliations:** 1 Department of Woman, Child and General and Specialized Surgery, University of Campania “Luigi Vanvitelli”, 80138 Naples, Italy; roberta.dellamarca@unicampania.it (R.D.M.); carla.zannella@unicampania.it (C.Z.); muhammad.shoaib1@unicampania.it (M.S.); shahab.bashir@unicampania.it (S.B.); massimiliano.galdiero@unicampania.it (M.G.); 2 Department of Chemistry, University of Bari Aldo Moro, Via Orabona 4, 70126 Bari, Italy; francesco.busto@uniba.it (F.B.); stefano.liotino@uniba.it (S.L.); maria.sportelli@uniba.it (M.C.S.); 3 INSTM, National Consortium of Materials Science and Technology, Via G. Giusti 9, 50121 Florence, Italy; 4 UOC Virology and Microbiology, University Hospital “Luigi Vanvitelli”, 80138 Naples, Italy

**Keywords:** green-synthesis, *Arctostaphylos uva-ursi*, silver nanoparticles (AgNPs), gellan gum/alginate films, antiviral properties

## Abstract

In recent years, nanotechnology has made remarkable progress in the fight against infectious diseases. However, the development of safe and effective antiviral drugs remains a challenge, as viruses rely on host cells for replication. Plant-derived, environmentally friendly nanoparticles have gained significant attention due to their low toxicity, which enables them to target viruses without damaging host cells. In this study, we describe the synthesis of silver nanoparticles (AgNPs) using *Arctostaphylos uva-ursi* leaf extract and explore their potential antiviral activity. The *uva-ursi* AgNPs were initially characterized using nanoparticle tracking analysis (NTA) and transmission electron microscopy (TEM). We then optimized two different gellan gum/alginate film formulations (1.6:0.4 and 1.2:0.8) as delivery matrices for the AgNPs and assessed Ag^+^ skin permeation using a Franz diffusion cell system. The antiviral potential of the *uva-ursi* AgNPs—both alone and incorporated into the films—was tested against herpes simplex virus type 1 (HSV-1). Our findings indicate that *uva-ursi* AgNPs may directly interact with the viral envelope, disrupting the lipid membrane and/or interfering with viral surface proteins. Overall, green-synthesized *uva-ursi* AgNPs may represent a natural, cost-effective, and safe alternative strategy for managing herpetic infections.

## 1. Introduction

Herpes simplex virus (HSV) infections are among the most prevalent human viral infections worldwide, primarily transmitted through direct person-to-person contact and responsible for a wide range of mucocutaneous manifestations [1].

Following primary infection, both herpes simplex virus type 1 (HSV-1) and type 2 (HSV-2) establish lifelong latency in sensory neurons, with the potential for periodic reactivation at or near the initial site of infection. While reactivation is often asymptomatic or results in mild recurrent lesions, HSV infections can occasionally lead to severe and potentially life-threatening complications, including herpetic keratitis, herpes simplex encephalitis, and neonatal herpes.

It is estimated that a substantial proportion of the global population is seropositive for HSV-1 and/or HSV-2, with prevalence rates approaching 90% in certain populations [2]. Despite the significant global burden and clinical impact of HSV infections, the development of an effective vaccine remains a major scientific challenge due to the virus’s ability to establish latency, evade host immune responses, and reactivate intermittently [3].

Current treatments largely rely on nucleoside analogs such as acyclovir, valacyclovir, and famciclovir, which specifically target viral DNA polymerase. However, these therapies are unable to eradicate latent infections and often prove less effective against drug-resistant strains, particularly in those with compromised immune systems [4]. Despite advancements in treatment, issues of drug resistance and side effects continue to be problematic [5]. As a result, there is growing interest in using nanotechnology-based antiviral strategies that may enhance efficacy, reduce the likelihood of resistance, and enable localized delivery [6]. Among the wide range of nanomaterials investigated, silver nanoparticles (AgNPs) have emerged as particularly promising due to their broad-spectrum antimicrobial activity, notable antiviral properties, and versatility in surface functionalization for diverse biomedical applications. In recent years, the use of nanoparticles in virology has attracted increasing interest, highlighting their potential to transform both antiviral therapeutic strategies and preventive interventions [7]. By leveraging the unique physicochemical properties of nanoparticles, researchers aim to develop targeted, efficient, and multifunctional approaches to combat viral infections [8]. Green synthesis methods for AgNPs are relatively simple, cost-effective, and environmentally friendly compared to conventional chemical synthesis methods. They typically involve the reduction of silver ions using natural reducing agents present in plant extracts or microbial cultures, making them easily scalable for large-scale production [9] without the presence of harmful chemical species or the production of toxic by-products. Previous studies described the utilization of several natural extracts in the synthesis of AgNPs [10,11]. For example, eco-friendly biogenic AgNPs, produced from sources such as *Penicillium* spp. or *Chlorella vulgaris*, also exhibited significant anti-HSV-1 activity in vitro, resulting in reduced viral titers and minimal cytotoxicity [12]. Furthermore, recent research has concentrated on integrating AgNPs into topical and mucosal delivery systems, such as chitosan gels, PLGA-(poly(lactic-co-glycolic) acid) based nanoparticles, and mucoadhesive hydrogels, to enhance localized antiviral effects, improve retention time, and reduce systemic toxicity [13,14]. In addition, AgNPs have attracted significant attention due to their broad-spectrum biological activities, including antimicrobial and anticancer effects. Their multifunctional properties suggest a strong potential as innovative nanomedicine tools for the prevention, diagnosis, and treatment of infections and cancer [15].

In this study, we employed the leaf extract of *Arctostaphylos uva-ursi* as a natural reducing agent for the synthesis of AgNPs. *Arctostaphylos uva-ursi* has a long-standing history in traditional herbal medicine due to its high concentration of hydroquinone glycosides (notably arbutin), tannins, and flavonoids that contribute to its robust antioxidant and antimicrobial properties [15,16,17,18,19], which has led to its clinical use in treating uncomplicated cystitis across Europe [20,21,22,23]. Despite these well-known properties, the application of *Arctostaphylos uva-ursi* extract in nanobiotechnology remains unexplored. Specifically, to date, no investigation has addressed the synthesis of AgNPs using this extract or evaluated their potential antiviral efficacy. Therefore, the primary aim of this work was to evaluate the feasibility of synthesizing AgNPs using *Arctostaphylos uva-ursi* leaf extract and to conduct a comprehensive physicochemical characterization using UV–Visible spectroscopy, nanoparticle tracking analysis (NTA), and transmission electron microscopy (TEM). As a further step, these nanoparticles were incorporated into a topical delivery system based on natural biopolymer hydrogels, which are of significant interest for skin applications due to their biocompatibility and ability to provide controlled drug release [24,25,26,27]. Building on our previous research [28], the study further aimed to develop an advanced topical delivery system by incorporating the synthesized AgNPs into hydrogel films based on gellan gum (GG) and sodium alginate (NaALG) blends at different polymer ratios (1.6:0.4 and 1.2:0.8). The thermal behavior of these formulations was analyzed through thermogravimetric analysis (TGA) and differential scanning calorimetry (DSC). Finally, the research focused on evaluating the performance of these formulations, specifically by analyzing silver ion skin permeation via Franz diffusion cells and determining the cytotoxicity and antiviral activity of the *uva-ursi* AgNPs—both alone and embedded in the hydrogel films—against herpetic infections. Through this integrated approach, the study proposes a novel, biocompatible platform for the topical treatment of viral infections.

## 2. Results

### 2.1. AgNPs Synthesis and Characterization

#### 2.1.1. Determination of AgNP Concentration Using Nanoparticle Tracking Analysis (NTA)

**Figure 1:**
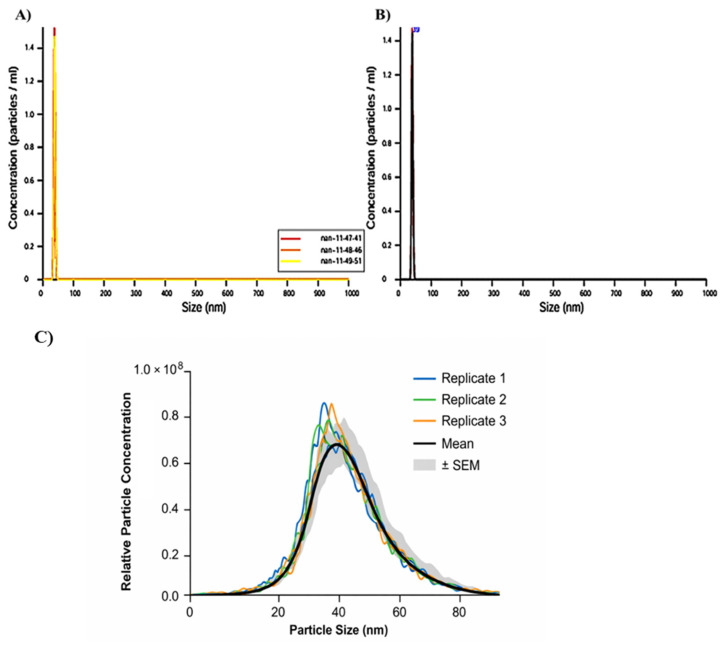
Nanoparticle tracking analysis (NTA) of AgNPs in a 1 mL sample. (**A**) Hydrodynamic diameter size per concentration, obtained using the finite track length adjustment (FTLA) algorithm, with triplicate measurements of the AgNPs sample. (**B**) Average of the three measurements. All measurements were run with non-diluted samples. (**C**) Relative particle concentration (particles/mL) is plotted as a function of particle size (nm). Three independent measurements are shown (Replicate 1–3; colored lines), together with the mean distribution (black line) and the shaded area representing ± standard error of the mean (SEM). The particle size distribution exhibits a unimodal profile, with the majority of particles in the 30–80 nm range. The data presented represent a normalized and scaled distribution derived from particle counts and do not correspond to absolute particle concentration values. The x-axis was rescaled to 0–100 nm to improve visualization of the size distribution.

To investigate the concentration and the range size of AgNPs in 1 mL of the sample, NTA analysis was executed. The AgNPs consisted of 8.6 × 10^8^ ± 2.10 × 10^6^ N° particles/mL, with an average diameter of 38.4 ± 0.3 nm and a mode value of 38.1 ± 0.3 nm (Figure 1).

#### 2.1.2. Morphological Characterization of AgNPs

**Figure 2:**
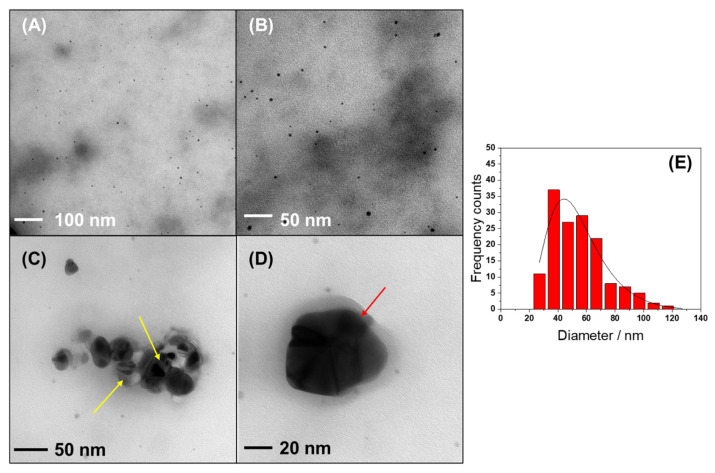
Morphological characterization of AgNPs prepared by *uva-ursi* by transmission electron microscopy (**A**–**D**). Size distribution histogram (**E**) (R^2^ = 0.89081) of AgNPs.

The morphological characterization of AgNPs prepared by *Arctostaphylos uva-ursi* extract was carried out by TEM. Typical micrographs of the synthesized particles are shown in Figure 2A–D and confirm the presence of spheroidal structures, having a log-normal distribution of measured diameters. Average particle size was equal to 46 ± 12 nm (Figure 2E).

The low-contrast background of low-magnification TEM micrographs is relevant to the presence of organic moieties coming from the bio-extract. These species seem to have a dispersing effect too: minor aggregation between particles is visible (Figure 2A,B). AgNPs probably have a nano-crystalline nature, showing interference fringes in high-magnification images (Figure 2C, yellow arrows). AgNPs are sterically stabilized by the *uva-ursi* extract: a low-contrast halo surrounding the particle (Figure 2D, red arrow) is attributed to the presence of the plant extract [29].

### 2.2. Hydrogel Film Characterization

#### 2.2.1. Swelling and Water Holding Capacity

**Figure 3:**
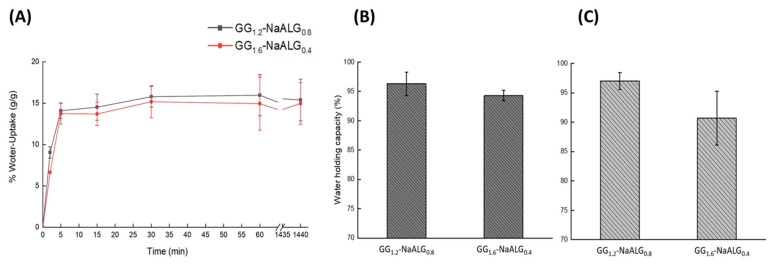
Water uptake (WU%) (**A**), and water holding capacity percentage (WHC%) of hydrogels in PBS (**B**) and SWF (**C**).

The swelling kinetics upon hydration were thoroughly investigated. As shown in Figure 3A, a plateau is reached after approximately 5 min, corresponding to about 14.0 ± 1.0% and 13.7 ± 1.0% for GG_1.2_-NaALG_0.8_ and GG_1.6_-NaALG_0.4_, respectively. The experiment was carried out at 32 °C in PBS, resulting in a rapid swelling that remained constant up to 24 h for both hydrogels. These results are in agreement with a previous study [30], in which GG- and NaALG-based hydrogels were employed as carriers for an olive leaf extract, aimed at providing protection against ultraviolet radiation generated by sunlight. In that case, the swelling kinetics of the GG_1.2-_NaALG_0.8_ and GG_1.6_-NaALG0_.4_ hydrogels were evaluated up to 60 min, while, in the present study, the water uptake behavior was observed up to 24 h. Regarding the water holding capacity test (WHC), the experiment was performed by dipping the hydrogel in PBS (pH =7.4) (Figure 3B) and SWF (Figure 3C). The results for both tests are nearly identical, since no significant differences (*p* < 0.05) were observed between the two buffers. Specifically, values of 96 ± 2% and 94 ± 1% were obtained in PBS and 97 ± 1% and 91 ± 5% in SWF for GG_1.2_-NaALG_0.8_ and GG_1.6_-NaALG_0.4_, respectively.

#### 2.2.2. DSC-TGA

**Figure 4:**
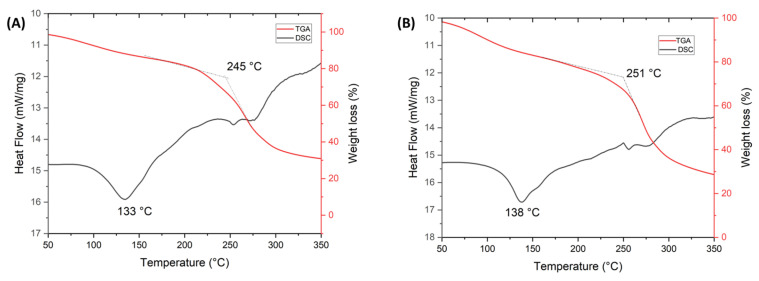
DSC (black line) and TGA (red line) curves of GG_1.2_-NaALG_0.8_ (**A**) and GG_1.6_-NaALG_0.4_ (**B**).

The films GG_1.2_-NaALG_0.8/_AgNPs (Figure 4A) and GG_1.6_-NaALG_0.4/_AgNPs (Figure 4B) were analyzed by DSC (black line) and TGA (red line). In the DSC curves, an endothermic peak associated with the melting process was observed, shifting from 133 °C to 138 °C as the gellan content increased. Moreover, the onset degradation temperature also increased from 245 °C to 251 °C. In the DSC thermograms, an increase in the released heat corresponded to the weight loss observed in the TGA curves.

### 2.3. Silver Release Evaluation

**Figure 5:**
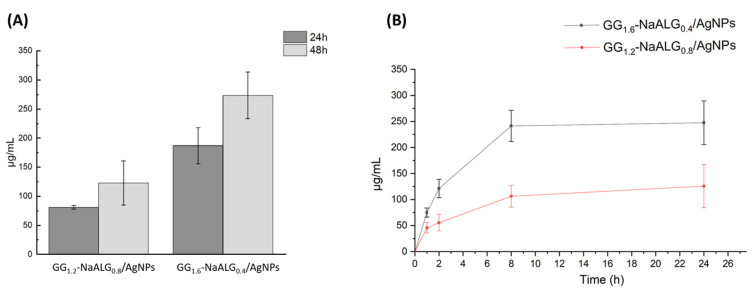
(**A**) Ag^+^ releases in PBS at 24 and 48 h, and (**B**) Ag^+^ release kinetics through a Franz diffusion cell until 24 h for AgNP-loaded hydrogel films.

In Figure 5, the Ag^+^ release profiles were evaluated for both AgNP-loaded hydrogel films. The experiments were conducted using two different setups: direct immersion in PBS, monitoring silver release at 24 and 48 h (Figure 5A), and the Franz diffusion cell system (Figure 5B), monitored up to 24 h. A similar release behavior between the two analyses was observed, as the hydrogel with the higher GG content released a greater Ag^+^ amount. For direct immersion times in PBS, Ag^+^ concentrations of 80.0 ± 3.0 µg/mL and 187.0 ± 30.0 µg/mL were registered after 24 h for GG_1.2_-NaALG_0.8_/AgNPs and GG_1.6_-NaALG_0.4_/AgNPs, respectively. These values increased at 48 h to 122.0 ± 40.0 µg/mL and 270.0 ± 45.0 µg/mL for the same samples (Figure 5A). A similar trend was observed in the Franz cell experiments, where concentrations of 126.0 ± 40.0 µg/mL and 247.0 ± 42.0 µg/mL were quantified for GG_1.2_-NaALG_0.8_/AgNPs and GG_1.6_-NaALG_0.4_/AgNPs, respectively (Figure 5B).

### 2.4. Cytotoxicity of AgNPs and AgNPs Film-Conjugated

**Figure 6:**
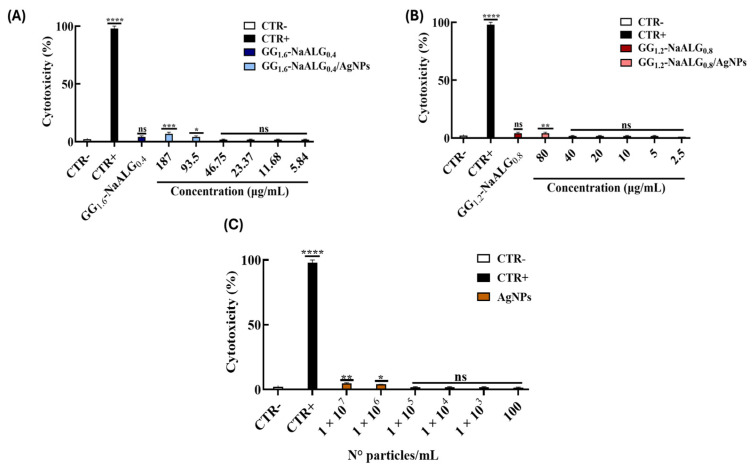
Evaluation of the two AgNP-containing films, GG_1.6_-NaALG_0.4_/AgNPs (**A**) and GG_1.2_-NaALG_0.8_/AgNPs (**B**), and of AgNP (**C**) cytotoxicity. Cytotoxicity was assessed in Vero-76 cells using an MTT assay after 24 h of treatment with the sample suspension. Blank films, without AgNPs, referred to as GG_1.6_-NaALG_0.4_ and GG_1.2_-NaALG_0.8_, were tested non-diluted. Data are reported as mean ± SD. CTR−: untreated cells; CTR+: 100% DMSO. Statistical analysis was performed using Dunnett’s multiple comparisons test: **** *p*-value < 0.0001; *** *p*-value = 0.0004; ** *p*-value = 0.0042; * *p*-value = 0.0358; ns *p*-value > 0.05.

The cytotoxic effects were evaluated on the Vero-76 cell line after 24 h of treatment with several samples: UU leaf extract, AgNPs and the two AgNP-containing films, GG_1.6_-NaALG_0.4_/AgNPs and GG_1.2_-NaALG_0.8_/AgNPs. As mentioned in paragraph 2.12, the GG_1.6_-NaALG_0.4_/AgNPs and GG_1.2_-NaALG_0.8_/AgNP-containing films, together with the blank films GG_1.6_-NaALG_0.4_ and GG_1.2_-NaALG_0.8_, were first immersed in PBS for 24 h before use. The two AgNP-containing films, GG_1.6_-NaALG_0.4_/AgNPs and GG_1.2_-NaALG_0.8_/AgNPs, were evaluated at concentrations of 187 μg/mL (Figure 6A) and 80 μg/mL (Figure 6B), respectively, using 2-fold serial dilutions. AgNPs were assessed starting from 1 × 10^7^ N° particles/mL (Figure 6C) in 10-fold dilutions. UU was examined at a concentration of 1.25 mg/mL. The two films GG_1.6_-NaALG_0.4_ and GG_1.2_-NaALG_0.8_ were overloaded with 0.9 mg of UU, immersed in 1 mL of PBS for 24 h, and tested the day after in 2-fold dilutions (Supplemental Materials, Figure S1). Blank films, without AgNPs, referred to as GG_1.6_-NaALG_0.4_ and GG_1.2_-NaALG_0.8_, were tested non-diluted.

As reported in Figure 6A–C, a negligible cytotoxic effect was observed for all samples analyzed at their highest concentrations, including UU and its corresponding films (Supplemental Materials, Figure S1).

In the light of these promising results, the antiviral activity was evaluated at concentrations that did not affect cell viability, starting from 5 x 10^6^ N° particles/mL for AgNPs, from 93.5 μg/mL for GG_1.6_-NaALG_0.4_/AgNPs and from 40 μg/mL for GG_1.2_-NaALG_0.8_/AgNP-containing films, respectively.

### 2.5. Antiviral Activity of AgNPs and AgNP-Conjugated Film

#### 2.5.1. Time-Shift Assays

**Figure 7:**
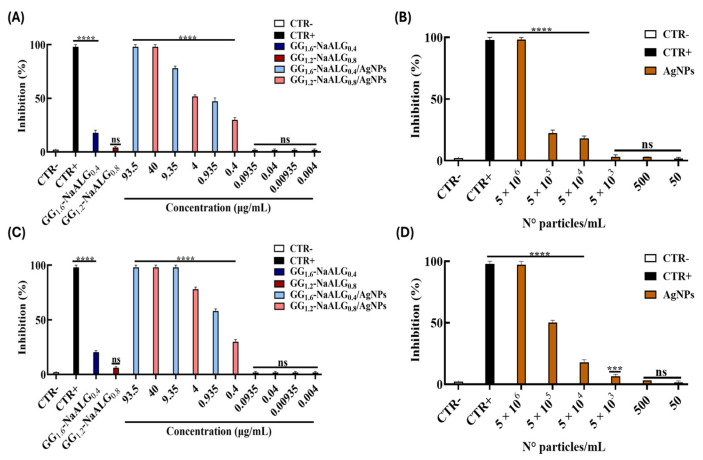
Time-shift assays of the two AgNP-containing films (**A**,**C**), GG_1.6_-NaALG_0.4_/AgNPs and GG_1.2_-NaALG_0.8_/AgNPs, and of AgNPs (**B**,**D**) to evaluate their potential antiviral effect against HSV-1. Two different plaque reduction assays are shown. (**A**,**B**) Co-treatment assay; (**C**,**D**) virus pre-treatment assay. The untreated cells represented the negative control (CTR−). Melittin (5 µM) was used as a positive control (CTR+) in the co-treatment and virus pre-treatment. Data are reported as mean ± standard deviation (SD) of three independent experiments. **** *p*-value < 0.0001; *** *p*-value < 0.0006; ns *p*-value > 0.05.

We conducted several plaque reduction assays. For the time-shift assays: in the co-treatment assay, the sample suspensions were co-incubated with the virus directly on the cellular monolayer (see Section 4.12 for more details). AgNP-conjugated film efficiently inhibited herpes infection. In particular, GG_1.6_-NaALG_0.4_/AgNPs recorded an IC_50_ of 1.392 µg/mL and GG_1.2_-NaALG_0.8_/AgNPs of 2.239 µg/mL, respectively (Figure 7A). Furthermore, the IC_50_ recorded by AgNPs alone was 9.1 × 10^5^ N° particles/mL (Figure 7B).

To investigate the mechanism of action of sample preparations, additional experiments were carried out. Virus pre-treatment is a set experiment in which the sample suspensions and virus were first incubated together at 37 °C for 1 h. The mixture was then diluted with a medium and titrated on the Vero cell monolayers. A more substantial inhibitory effect was observed by pre-incubating the virus with AgNP-conjugated film, showing an IC_50_ value of 0.7820 µg/mL for GG_1.6_-NaALG_0.4_/AgNPs and of 1.018 µg/mL for GG_1.2_-NaALG_0.8_/AgNP-containing films, respectively (Figure 7C). Even AgNPs performed a greater inhibitory effect in this experimental setting (IC_50_ of 3.9 × 10^5^ N° particles/mL, Figure 7D). Subsequently, the Vero cells were pre-treated with both AgNPs or AgNP-conjugated films for 1 h before infection (cell pre-treatment assay, Supplemental Materials, Figure S2A,B); alternatively, the cell monolayer was infected for 1 h and then treated with AgNPs and AgNP-conjugated film (post-treatment assay, Supplemental Materials, Figure S2C,D). In both cases, any sample suspensions did not have an inhibitory effect, suggesting that any preparation was able to stop infection by interacting with the cell membrane and was not able to reach the cell’s internal compartment or to influence the virus replication once inside the cell. However, the promising results in the virus pre-treatment setting suggested that AgNPs, both alone and film-associated, could act in the extracellular phase of infection, most likely targeting the viral envelope. 

To verify whether the AgNPs alone and AgNP-conjugated film have an enhanced inhibitory effect compared to the leaf extract itself, or to UU-conjugated film, GG_1.6_-NaALG_0.4_/uva ursi and GG_1.2_-NaALG_0.8_/uva ursi, we employed the same experimental settings for the latter as reported in Supplemental Materials, Figure S3. We found that even if UU, GG_1.6_-NaALG_0.4_/uva ursi and GG_1.2_-NaALG_0.8_/uva ursi showed a remarkable effect in the virus pre-treatment assay (IC_50_ values between 38 and 55 µg/mL; see Supplemental Materials Table S1), the effectiveness of the AgNP-conjugated films remains significantly higher, suggesting their crucial interaction within the viral particles.

#### 2.5.2. Temperature-Shift Assays

**Figure 8:**
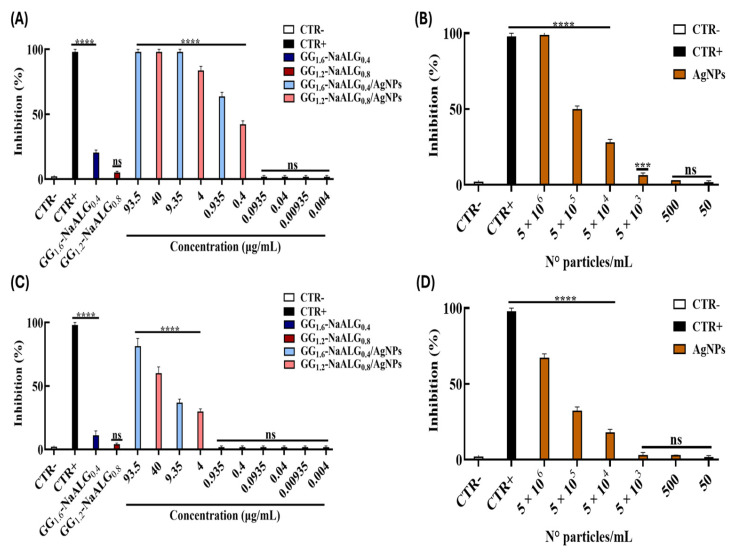
Temperature-shift assays of the two AgNP-containing films (**A**,**C**), GG_1.6_-NaALG_0.4_/AgNPs and GG_1.2_-NaALG_0.8_/AgNPs, and of AgNPs (**B**,**D**) to evaluate their potential antiviral effect against HSV-1. Two different plaque reduction assays are shown. (**A**,**B**) Attachment assay; (**C**,**D**) entry assay. Untreated cells served as the negative control (CTR−), while heparin (1 mg/mL) was used as the positive control (CTR+) in both assays. Data are reported as mean ± standard deviation (SD) of three independent experiments. Statistical significance was determined as follows: **** *p*-value < 0.0001; *** *p*-value < 0.0006; ns *p*-value > 0.05.

Temperature-shift assays were carried out to assess whether AgNPs were active during the virus attachment or the entry phase (Figure 8). An attachment assay evaluated the ability of the AgNPs to interfere with the virus binding to the target cell, but not its entry (Figure 8A). Alternatively, an entry assay is useful to evaluate the ability of the AgNP preparations to interfere with the intracellular entry of HSV-1 (Figure 8B).

As shown in Figure 8A,B, AgNPs, alone or film-associated, could interact during the virus adhesion in a dose-dependent manner with an inhibition rate that reflected the one observed in the virus pre-treatment assay. IC_50_ values of 0.6116 µg/mL for GG_1.6_-NaALG_0.4_/AgNPs and 0.6971 µg/mL for GG_1.2_-NaALG_0.8_/AgNPs were the lowest recorded in all the plaque reduction assays performed. The same scenario occurred for AgNPs alone, with an IC_50_ recorded of 2.9 × 10^5^ N° particles/mL. On the other hand, the sample suspensions slightly interfered with the viral entry phase (Figure 8C,D).

As for the time-shift assays, we evaluated these findings in comparison with the leaf extract and its films (Supplemental Materials, Figure S4). In this instance as well, UU, itself and film-associated, demonstrated superior activity in the attachment assay among all the antiviral tests conducted (IC_50_ values ranging from 28 to 37 µg/mL, Supplementary Materials, Table S1); however, as previously reported, the most promising outcomes are confirmed to be linked to the film-associated AgNPs.

In summary, Table 1 reports IC_50_ values for each antiviral assay presented here. IC_50_ values were derived from the attachment assay, as it represents the most relevant condition for evaluating interference with the early stages of viral infection. The selectivity index (SI) was estimated using the highest non-cytotoxic concentration tested (SI > max non-cytotoxic concentration tested/IC_50_). In detail, as the highest non-cytotoxic concentration, we have considered 93.5 and 40 µg/mL for GG1.6-NaALG0.4/AgNPs and GG1.2-NaALG0.8/AgNPs, respectively, and 5 × 10^6^ particles/mL for AgNPs in suspension. Notably, GG1.6-NaALG0.4/AgNPs and GG1.2-NaALG0.8/AgNPs exhibited SI values >150 and >57, respectively, while AgNPs alone showed SI >17, indicating a favorable therapeutic window.

### 2.6. Viral Gene Expression Analysis (Real-Time PCR) of GG_1.6_-NaALG_0.4_/AgNPs

**Figure 9:**
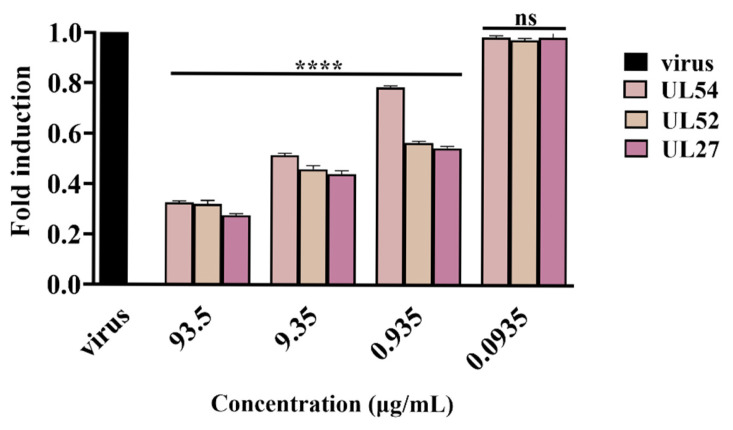
Viral gene expression analysis. Real-time PCR was performed to evaluate the effect of GG_1.6_-NaALG_0.4_/AgNP-conjugated film viral gene expression. For HSV-1, the expression levels of immediate early (UL54), early (UL52), and late (UL27) genes were analyzed. “Virus” refers to infected but not untreated cells**.** Data are reported as mean ± standard deviation (SD) of three independent experiments. Statistical significance was determined, comparing treated samples to ctr-, as follows: **** *p*-value < 0.0001, ns *p*-value > 0.05.

Given that the GG_1.6_-NaALG_0.4_/AgNP-conjugated film demonstrated the most effective inhibition profile in the plaque reduction tests, a qPCR was conducted to verify its potential efficacy (Figure 9). In detail, UL54 is an immediate early gene encoding the ICP27 protein involved in HSV-1 replication by inhibiting mRNA splicing at the post-transcriptional level and promoting the export of viral transcripts; UL52 is an early gene encoding DNA primase, and the UL27 gene represents a late gene coding the structural glycoprotein B (gB) [31]. A virus pre-treatment assay was performed, RNA was collected after 24 h, reverse-transcribed into cDNA, and amplified by real-time PCR.

The data revealed that viral infection decreased in a dose-dependent manner, indicating the virucidal efficacy of GG_1.6_-NaALG_0.4_/AgNP-conjugated film (Figure 9). As the concentration of GG_1.6_-NaALG_0.4_/AgNPs decreased, the expression of viral genes increased, eventually reaching levels comparable to those in the virus control. These findings, together with the results of the virus pre-treatment and attachment assays, suggest that AgNPs may interfere with early stages of viral infection, likely affecting viral attachment and/or entry. However, direct evidence of the underlying molecular mechanism is beyond the scope of the present study.

## 3. Discussion

HSV infection is a complex, multi-step process that begins with viral adhesion to the host cell, followed by entry, replication, assembly, and eventual egress. The initial stages, adhesion and entry, are critical for establishing infection. Recent studies have investigated the antiviral potential of AgNPs, particularly those synthesized using plant extracts, as a novel strategy to block HSV infections [32,33]. These phyto-fabricated AgNPs combine the inherent antiviral properties of silver with the bioactive phytochemicals from the plant extract, often resulting in synergistic effects [34]. One of the most promising aspects of AgNPs is their ability to interfere with the early stages of viral infection, particularly viral entry. Studies have shown that AgNPs can bind to viral surface proteins, such as glycoprotein B and D, thereby preventing their interaction with host cell receptors [35]. In the same way, surface-modified AgNPs, such as those functionalized with tannic acid (TA-AgNPs), exhibited strong anti-HSV-2 effects. These ~33 nm particles prevented viral attachment and entry, significantly decreasing genital lesions in mouse models, while also boosting adaptive immune responses [12]. Another study involved sulfonate-modified AgNPs (Ag-MES), which mimic heparan sulfate proteoglycans, the natural receptors for HSV entry. These particles demonstrated selective inhibition of HSV-1 entry into host cells with low cytotoxicity [36].

Following these studies, our research presents the synthesis and characterization of AgNPs from plant extracts. In particular, we propose AgNPs embedded in GG/NaALG films as an innovative strategy for skin dressings, showcasing their potential for topical prophylaxis or therapeutic applications.

HSV-1 infection can cause laceration of the skin tissue [37]. Indeed, to promote tissue healing, adequate absorption of exudate is necessary [38]. Therefore, the use of hydrogels can be considered a promising strategy for the treatment of wounds caused by HSV-1 skin infections [35]. For this reason, it was necessary to evaluate the swelling and water-holding capacity of the hydrogels, simulating the application method on the wounds, both in terms of pH and chemical composition, using PBS and SWF [28]. High water uptake and WHC values are essential characteristics for hydrogel-based biomaterials used in wound dressings. These properties enable the hydrogel to effectively absorb wound exudate, maintaining a moisturized environment that promotes wound healing, supports tissue regeneration, and reduces the risk of infection [39,40]. For these reasons, based on the results reported in Figure 3, we positively assessed that the proposed hydrogel films (despite the different GG and NaALG ratios) are a promising strategy for exudate retention. As shown in Figure 5A,B, a higher Ag^+^ release for the films with a higher gellan content was observed, both for direct release in PBS and using the Franz diffusion cell. Among the two formulations, GG_1.6_-NaALG_0.4_/AgNPs released a higher amount of silver ions, which correlates with its consistently superior antiviral performance across all assays, as we will discuss later in the text. The release kinetics study using a Franz diffusion cell was carried out to simulate a potential transdermal release of Ag^+^, as well as to assess the amount of Ag^+^ remaining entrapped in the StratM^®^ membrane.

The shift in the endothermic peak observed in Figure 4A,B is consistent with previous studies [28,41], which confirm that a higher gellan content leads to an increase in melting temperature. This increase can be attributed to an enhancement in the crystallinity of NaAlg induced by the presence of gellan [42]. The incorporation of gellan gum likely reinforces the conformation of the amorphous chains in the blend films, reducing the free volume compared to pure NaALG films, which exhibit a melting temperature of 116 °C [41]. Moreover, the presence of a single melting peak suggests good interaction between the two polymers in both blends. In addition, the onset degradation temperature also increases, in agreement with previous reports [43], indicating that higher gellan content improves the thermal stability of the material.

For all the biological experiments, the GG_1.6_-NaALG_0.4_/AgNPs and GG_1.2_-NaALG_0.8_/AgNP-containing films were first immersed in phosphate-buffered saline solution (PBS, pH 7.4) for 24 h before use. In our in vitro model (Figure 6), a negligible cytotoxic effect was observed for the two AgNP-containing films, GG_1.6_-NaALG_0.4_/AgNPs and GG_1.2_-NaALG_0.8_/AgNPs, as well as for AgNPs alone at their highest concentration. Although cytotoxicity was evaluated only in Vero-76 cells, a well-established model for HSV-1 infection, we acknowledge that the use of a single cell line represents a limitation of the study. Nevertheless, the obtained results provided an opportunity for further investigations.

Antiviral assays revealed that the AgNP-loaded films, GG_1.6_-NaALG_0.4_/AgNPs and GG_1.2_-NaALG_0.8_/AgNPs, strongly inhibited HSV-1 infection, particularly during co-treatment and, even more prominently, during virus pre-treatment (Figure 7A,C). Their ability to significantly reduce viral infectivity when incubated directly with the virus suggests a possible mechanism involving interaction with viral particles. This finding was primarily supported by the data of the attachment assay (Figure 8A,B) in which viral adhesion appears to be reduced in a dose-dependent manner, and the IC_50_ values recorded were 0.6116 µg/mL for GG_1.6_-NaALG_0.4_/AgNPs, 0.6971 µg/mL for GG_1.2_-NaALG_0.8_/AgNPs, respectively, and 2.9 × 10^5^ N° particles/mL for AgNPs alone. The estimation of the SI further supports the favorable safety profile of the developed system. Notably, the AgNP-loaded films exhibited high SI values (>150 and >57), indicating a strong antiviral effect at concentrations well below those associated with cytotoxicity. Between the two films, GG_1.6_-NaALG_0.4_/AgNPs film-associated showed the best inhibitory profile. The higher proportion of gellan gum in this formulation likely creates a more hydrated and porous network, facilitating ion diffusion and enabling a more efficient interaction of Ag^+^ or AgNPs with viral particles. This correlation is in line with previous evidence showing that the antiviral potency of silver-based systems strongly depends on the availability of ionic silver or surface-reactive AgNPs, which have been reported to interact with viral envelopes or glycoproteins, potentially leading to structural alterations and reduced infectivity [44]. For this reason, the GG_1.6_-NaALG_0.4_/AgNPs suspension was further investigated for its antiviral activity through a real-time PCR. As shown in Figure 9, the expression levels of HSV-1 immediate early (*UL54*), early (*UL52*), and late (*UL27*) genes were evaluated after a virus pre-treatment assay in a concentration range of 0.0935–93.5 µg/mL. The data revealed that viral infection decreased in a dose-dependent manner, supporting its potential virucidal efficacy. In general, AgNPs alone displayed a similar trend, although the film-conjugated forms consistently outperformed free nanoparticles, highlighting the beneficial impact of the polymeric matrix. For instance, tartaric acid, one of the film’s main constituents, in combination with other compounds, has been reported for its antiviral potential against HSV-1 and HSV-2 [45], thereby enhancing the suitability of our films for treating skin infections. The comparison with the *uva-ursi* extract and the UU-loaded films revealed that, although these preparations also exert inhibitory activity, the AgNP-loaded films remain substantially more potent. The synergy between AgNPs and the GG/NaALG matrix may further enhance nanoparticle availability, dispersion, or affinity for the viral surface.

In summary, our approach highlights the encouraging prospects of the biogenic AgNPs as innovative antiviral agents targeting HSV-1, which could be utilized for topical antiviral treatments. Continuous refinement of the NP composition, surface modification, and delivery methods will be essential to fully unlock their clinical potential. However, further studies will be necessary to directly elucidate the underlying mechanism of antiviral action.

## 4. Materials and Methods

### 4.1. Materials

*Arctostaphylos Uva-Ursi* leaf extract, coded as UU, containing total hydroquinone derivatives expressed as anhydrous arbutin in the range 19.0–21.0% *_w_*_/*w*_, was purchased from Farmalabor S.p.A. (Apulia, Italy). Low acyl content gellan gum (Phytagel™, molecular weight 1 × 10^6^ g/mol)—coded as GG—was purchased from Sigma-Aldrich (Merck, Milan, Italy). Sodium alginate (Cat #180947, Sigma-Aldrich, St. Louis, MO, USA, viscosity 15–25 cP, 1% in H_2_O), coded as NaALG_,_ was purchased from Sigma-Aldrich (Merck, Milan, Italy). Tartaric acid and glycerol were purchased from Sigma-Aldrich (Merck, Milan, Italy). Films were prepared with ultrapure water, obtained through a Milli-Q^®^ distillation system (Millipore-Merck, Darmstadt, Germany).

HNO_3_ (67%, Trace-SELECT^®^ Ultra, for ultratrace analysis), NaH_2_PO_4_ (Trace-SELECT^®^, ≥99.99%, for trace analysis), and Na_2_HPO_4_ (Trace-SELECT^®^, ≥99.99%, for trace analysis) were from Merk Sigma-Aldrich^®^. Water for trace analysis at ppt level (Water RS—Ultrapure) was purchased from CARLO ERBA Reagents SAS^®^.

### 4.2. Preparation of AgNPs from Actostaphylos uva-ursi Leaf Extract

**Scheme 1:**
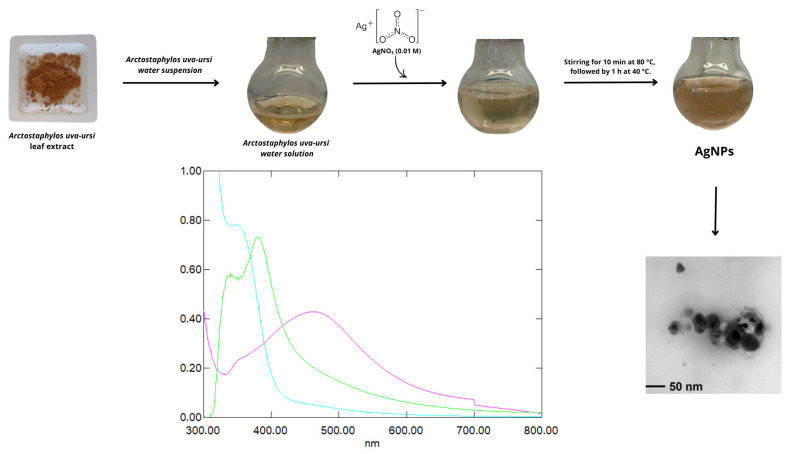
Schematic representation of the *uva-ursi* AgNP preparation steps and the UV-Vis absorption spectra of AgNO_3_ solution (light blue line), *Arctostaphylos uva-ursi* extract (green line), and *uva-ursi* AgNPs (fuchsia line).

The AgNPs synthesis method was adapted from Thirumoorthy et al. with some modifications [46]. *Arctostaphylos uva-ursi* extract (0.006 g/mL) was centrifuged at 4000 rpm, and the resulting supernatant was collected and diluted 1:2. This solution (10 mL) was transferred to a round-bottomed flask and gently shaken at 80 °C for 10 min under dark conditions. Subsequently, 10 mL of AgNO_3_ (0.01 M) was added dropwise to the mixture. The reaction was maintained under stirring for 10 min at 80 °C, followed by 1 h at 40 °C. The yield of the synthesis, expressed as the amount of total silver that has been reduced to form nanoparticles, was equal to 72 ± 9%. The different steps for AgNP preparation are reported in Scheme 1, along with the UV-Vis spectra of the starting materials and nanoparticles, which allowed us to verify their formation by observing the surface plasmon resonance (SPR) band at 460 nm [29].

The stability of AgNPs was evaluated spectrophotometrically over a period of 5 days. The UV–Vis absorption spectra remained unchanged and highly reproducible compared to freshly prepared samples, confirming the colloidal stability of the nanoparticles within this period.

### 4.3. Determination of AgNP Concentration by NTA

The concentration of *uva-ursi* AgNPs was determined using a NanoSight NS300 NTA instrument (Malvern Panalytical, Great Malvern, UK). In brief, 1 mL of *uva-ursi* AgNPs was inserted into the instrument with a syringe pump speed of “30” and a level chamber of “3”. Three readings were taken at room temperature (21.5 °C). 1498 frames in total were analyzed by NTA software (Malvern Instruments, version 3.4, Worcestershire, UK) using a detection threshold of 5.

### 4.4. Morphological Characterization of AgNPs by TEM

AgNPs were studied using transmission electron microscopy (TEM), using a FEI Tecnai 12 instrument (120 kV; filament: LaB_6_, Thermo Fisher Scientific, Waltham, MA, USA). Colloidal samples were deposited by drop-casting (2–3 μL) onto carbon-coated Cu grids (300 mesh, Agar Scientific Ltd., Rotherham, UK). The microscope was calibrated by using the S-106 Cross Grating (2160 lines/mm, 3.05 mm) supplied by Agar Scientific. Alignments and astigmatism correction were carried out following factory settings and fast Fourier transform (FFT) processing, respectively. The size distribution histogram was obtained from micrograph processing, using the ImageJ (version 1.8.0) software, measuring at least 250 particles. All analytical data were treated and plotted using Origin Pro^©^ 2022 software.

### 4.5. Hydrogel Film Preparation

Film preparation was upgraded, starting from previously tested GG/NaALG ratios [29], maintaining the sum of the two polymer percentages equal to 2% *_w_*_/*v*_. Two different GG:NaALG ratios were considered: GG 1.6% _*w*/*v*_ and NaALG 0.4% _*w*/*v*_ (GG_1.6_-NaALG_0.4_) and GG 1.2% _*w*/*v*_ and NaALG 0.8% _*w*/*v*_ (GG_1.2_-NaALG_0.8_). Tartaric acid at 0.2% _*w*/*v*_ was initially solubilized in distilled water at 80 °C, followed by the polymer powder addition under continuous stirring, on a magnetic plate (VELP Scientifica Srl, Usmate, Italy). Glycerol at 1% _*w*/*v*_ was finally added to the solution as a plasticizer. When AgNP-loaded films were produced, 2 × 10^7^ NPs/mL was added to the solution after lowering the temperature to 40 °C. Smooth and homogeneous film-forming solutions were cast on Petri dishes polystyrene, 90 mm diameter (Sigma-Aldrich, St. Louis, MO, USA) and dried in an oven (PID system, MPM Instruments S.r.l., Bernareggio, Italy) at 40 °C for 24 h. Blank films, without AgNPs, were also prepared for comparison. In Table 2, a summary of the different films prepared is reported.

### 4.6. Hydrogel Film Swelling and Water Holding Capacity

Dry hydrogel samples were cut into square pieces with 1 cm^2^ area, accurately weighed (m_i_^d^), and immersed in phosphate-buffered saline solution (PBS, pH 7.4) and simulated wound fluid (SWF) prepared as reported by Svensby et al. [47], at 32 °C to determine the swelling kinetics up to 24 h. All the specimens were weighed after each time point (m_i_^t^). The water uptake (expressed as grams of PBS per gram of dry film) was calculated using the following formula (Equation (1)):

(1) Water uptake=(m_i^t−m_i^d)/(m_i^d)

Moreover, for water holding capacity measurements, the hydrated hydrogel films were centrifuged in a centrifuge equipped with a basket rotor able to deliver 1400 rpm. The centrifuged samples were weighed, and the retained water was expressed as a weight percentage of the total uptake of water (Equation (2)):

(2) Water Holding Capacity((m_c−m_d))/((m_s−m_d))×100

where m_c_, m_s_, and m_d_ are the weights of centrifuged, swollen, and dry hydrogels.

### 4.7. In Vitro Skin Permeation Studies

The in vitro skin permeation studies were performed using a Franz diffusion cell (PermeGear Inc., SES GmbH, Bechenheim, Germany) using the protocol reported in a previous work [29], with some modifications. Briefly, AgNP-loaded hydrogel films (about 1 cm^2^) were placed in the cell donor compartment. An O-ring joint kept the film (0.6 cm^2^) on the synthetic StratM^®^ membrane (Merck KGaA, Darmstadt, Germany), characterized by skin-like porosity, diffusivity, and composition. The whole assembly was fixed with a stainless-steel clamp to maintain the tight connection between the donor and receptor compartments. A total of 1 and 5 mL of PBS (pH 7.4) were added to the donor and acceptor compartments, respectively. In the acceptor compartment, the buffer solution was continuously stirred using an ATE magnetic stirrer (VELP Scientifica Srl, Usmate, Italy). The temperature was kept constant at 32.00 ± 0.03 ^°C^, using a CD-B5 heating circulator bath (Julabo GmbH, Seelbach, Germany). To evaluate the Ag^+^ release kinetics, at predetermined time points (1, 2, 8, and 24 h), PBS aliquots of 500 μL were withdrawn, replaced with fresh buffer, and analyzed by electrothermal atomic absorption spectroscopy (ETAAS) as described in Section 2.3. At the end of the experiment, the StratM^®^ was sectioned with a scalpel into six parts and placed into 5 mL of PBS, mixed with Vortex (VELP Scientifica Srl, Usmate, Italia) for 1 min, and left overnight at 25 °C to extract and quantify the Ag^+^ retained by the membrane.

### 4.8. DSC-TGA

The thermal behavior of the films was investigated by differential scanning calorimetry (DSC) and thermogravimetric analysis (TGA).

DSC was carried out using a Perkin–Elmer DSC 7 calorimeter (Perkin-Elmer Inc., Waltham, MA, USA), putting samples into aluminum pans. The scans were performed at a rate of 10 °C/min, from 50 to 350 °C, in a N_2_-saturated atmosphere.

TGA was performed on a Perkin Elmer TGA-400 instrument (Perkin Elmer Inc., Waltham, MA, USA), heating 5–10 mg of samples in a N_2_-saturated atmosphere and setting the gas flow to 20 mL/min. The flow rate was set to 20 °C/min, heating from 50 °C to 350 °C. For each sample, thermograms (TG) with respective derivative (DTG) curves were recorded and analyzed with the TGA Pyris software.

### 4.9. Silver Ionic Release Evaluation by Atomic Absorption Spectroscopy

Ag^+^ ion release kinetics were determined by electrothermal atomic absorption spectroscopy (ETAAS). Samples were cut into 1 cm^2^ squares and soaked in Eppendorf^®^ tubes (Sigma-Aldrich, St. Louis, MO, USA) containing 1 mL of PBS contact solution (pH = 6.8, ionic strength I = 0.1). This pH resembles the one used for antiviral experiments. At definite times, contact solution was sampled and properly diluted/mineralized with 0.2% *_w_*_/*v*_ HNO_3_ to undergo ETAAS analysis. We used a Perkin Elmer PinAAcle AS900Z spectrometer, equipped with a Ag hollow cathode lamp and a transversely heated graphite furnace. A calibration curve was accomplished using proper dilutions of a Ag^+^ standard solution for AAS. Bare films (i.e., without AgNPs) were used as controls. All experimental points were plotted using Origin Pro^©^ 2022 software.

### 4.10. Cell Line and Viral Strains

*Cercopithecus aethiops* kidney cells (Vero-76, ATCC CRL 1587) were purchased from the American Type Culture Collection (ATCC, Manassas, VA, USA). Cells were cultured in Dulbecco’s Modified Eagle Medium (DMEM, Microtech, Naples, Italy) with 4.5 g/L of glucose (Microtech, Naples, Italy), 2 mM of L-glutamine (Microtech), 100 IU/mL of penicillin-streptomycin solution (Himedia, Naples, Italy) and supplemented with 10% fetal bovine serum (FBS, Microgem, Naples, Italy) in a humidified atmosphere with 5% CO_2_ at 37 °C. As previously reported, HSV-1 (strain SC16) was propagated on Vero-76 cells [48].

### 4.11. Cytotoxicity Assay

For all the biological experiments that follow, the GG_1.6_-NaALG_0.4_/AgNPs and GG_1.2_-NaALG_0.8_/AgNP-containing films, together with the blank films GG_1.6_-NaALG_0.4_ and GG_1.2_-NaALG_0.8_, were first immersed in phosphate-buffered saline solution (PBS, pH 7.4) for 24 h before use.

The cytotoxicity effect of *uva-ursi* leaf extract, AgNPs, GG_1.6_-NaALG_0.4_/AgNPs, and GG_1.2_-NaALG_0.8_/AgNPs was evaluated through the 3-(4,5-dimethythiazol-2-yl)-2,5-diphenyltetrazole bromide (MTT) assay. A density of 2 × 10^4^ cells/well was seeded in 96-well plates, and the day after, the preparations were tested as follows: GG_1.6_-NaALG_0.4_/AgNPs at 187 μg/mL and GG_1.2_-NaALG_0.8_/AgNPs at 80 μg/mL, in 2-fold serial dilutions, respectively. AgNPs were tested from 1 × 10^7^ N°particles/mL in 10-fold dilutions. UU was tested at 1.25 mg/mL. The two films GG_1.6_-NaALG_0.4_ and GG_1.2_-NaALG_0.8_ were overloaded with 0.9 mg of UU, immersed in 1 mL of PBS for 24 h, and tested the day after in 2-fold dilutions (Supplementary Materials, Figure S1). Blank films, without AgNPs, referred to as GG_1.6_-NaALG_0.4_ and GG_1.2_-NaALG_0.8_, were tested non-diluted. Untreated and treated cells with 100% DMSO represented CTR− and CTR+. After 24 h of exposure, 100 μL of MTT solution was added to each well for 3 h at 37 °C, then the solution was solubilized, and the absorbance was read at 570 nm using a microplate reader (Tecan, Männedorf, Switzerland). The cytotoxicity percentage was calculated according to the following formula (Equation (3)):

(3) % Cytotoxicity=100−[ 100×(OD 570 nm of the test sample/OD 570 nm of CTR−)]

### 4.12. Antiviral Activity

The antiviral potential of UU leaf extract, AgNPs, GG_1.6_-NaALG_0.4_/AgNPs, and GG_1.2_-NaALG_0.8_/AgNPs was investigated. Different plaque-reduction assays were conducted as previously reported [49]. Vero-76 cells were seeded in 24-well plates (1.2 × 10^5^ cells/well) and grown for 24 h at 37 °C in 5% CO_2_. Samples were tested as follows: GG_1.6_-NaALG_0.4_/AgNPs at 93.5 μg/mL and GG_1.2_-NaALG_0.8_/AgNPs at 40 μg/mL in 10-fold serial dilutions, respectively. AgNPs were tested from 5 × 10^6^ N°particles/mL in 10-fold dilutions. Uva ursi was tested at concentrations ranging from 625 μg/mL in a 10-fold serial dilution. The two films GG_1.6_-NaALG_0.4_ and GG_1.2_-NaALG_0.8_ were overloaded with 0.9 mg of UU, immersed in 1 mL of PBS for 24 h, and tested the day after from 450 μg/mL in 10-fold dilutions (Supplementary Materials). Blank films, GG_1.6_-NaALG_0.4_ and GG_1.2_-NaALG_0.8_, were tested non-diluted. The viral strain was used at a multiplicity of infection (MOI) of 0.01. After treatment, the monolayer was washed, covered with the complete medium (10% FBS) supplemented with 3% carboxymethylcellulose (CMC) (Sigma-Aldrich, C5678, C5013, St. Louis, MO, USA), and incubated for 48 h at 37 °C and 5% CO_2_. Finally, cells were fixed with 4% formaldehyde (Sigma-Aldrich, St. Louis, MO, USA) and stained with crystal violet solution. For each sample, the antiviral assays were performed as follows [49].

#### 4.12.1. Time-Shift Assays

(i) Co-treatment assay: Sample suspensions and virus were simultaneously incubated on Vero-76 cells for 1 h at 37 °C. (ii) Cell pre-treatment assay: First, the sample, in its concentration range, was added to Vero-76 cells for 1 h. Then, it was removed, and cells were infected with viral suspensions for 1 h at 37 °C. (iii) Virus pre-treatment assay: Sample suspensions and virus were incubated together for 1 h at 37 °C at an MOI of 0.1 pfu/mL. This initial MOI was used to promote effective interaction between viral particles and the tested compounds during the pre-incubation step, ensuring sufficient virus–compound contact while allowing the antiviral agent to be diluted to a concentration that is not active per se in standard antiviral conditions. Following this incubation, the mixture (sample + virus) was diluted to a final MOI of 0.01 before infection. This dilution was performed to achieve optimal plaque resolution, avoid saturation effects, and enable accurate and reliable quantification of antiviral activity. Dilutions were added to the Vero-76 monolayer for 1 h at 37 °C and 5% CO_2_. (iv) Post-treatment assay: Cells were previously infected with the viral suspension in DMEM w/o FBS for 1 h at 37 °C. Then, the cell monolayer was washed to remove extracellular virions and treated with the sample suspensions for 1 h at 37 °C.

#### 4.12.2. Temperature-Shift Assays

(i) Entry assay: Pre-cooled Vero-76 cells were first infected for 1 h at 4 °C to permit viral attachment. Then, cells were washed and treated with sample suspensions for 1 h at 37 °C. After the treatment, cells were covered with CMC and incubated for 48 h at 37 °C and 5% CO_2_. (ii) Attachment assay: Pre-cooled Vero-76 cell monolayers were infected in the presence of samples for 1 h at 4 °C. Subsequently, cells were washed to remove unabsorbed viruses, overlaid with CMC, and incubated for 48 h at 37 °C and 5% CO_2_.

The untreated cells represented the negative control (CTR−). At the same time, several compounds were used as positive controls (CTR+): melittin (5 µM) in the co-treatment and virus pre-treatment, dextran-sulfate (1 µM) in cell pre-treatment, and aciclovir (5 µM) in post-treatment [49]; heparin (1 mg/mL) for the attachment and entry assays [50].

The inhibition rate of viral infectivity was calculated by comparing the number of plaques counted in cells treated with sample suspensions to the plaques detected in the CTR−, according to the following formula (Equation (4)):

(4) %Viral Inhibition=100−[ 100 ×(plaques counted in the test sample/plaques counted in the CTR−)]

The 50% inhibitory concentration (IC_50_) was calculated by linear regression analysis.

### 4.13. Viral Gene Expression Analysis (Real-Time PCR)

Since the GG_1.6_-NaALG_0.4_/AgNPs sample showed the best inhibition profile in the plaque reduction assays, a qPCR was performed to confirm its promising activity. The virus pre-treatment scheme was conducted as described above. After 30 h of incubation, the total RNA was extracted from the cells using TRIzol reagent (Thermo Fisher, Waltham, MA, USA). 1 µg of the obtained RNA was retrotranscribed into cDNA, according to the instructions of 5× All-In-One RT MasterMix (Applied Biological Materials, Richmond, BC, Canada). The qPCR was performed in a total volume of 20 µL containing 0.1 µM of each primer, BrightGreen 2× qPCR MasterMix-No Dye (Applied Biological Materials), and 100 ng of cDNA. Amplification was conducted in Thermal Cycler UNO96 (VWR International, Radnor, PA, USA) using the following amplification program: denaturation at 95 °C for 15 s, annealing at 60 °C for 60 s, and extension at 72 °C for 20 s (40 cycles). The expression of specific viral genes *UL54* (immediate early gene), *UL52* (early gene), and *UL27* (late gene) was evaluated using the primer sequences (Euforins, Milan, Italy) reported in Table 3.

The target threshold cycle (Ct) values were normalized to glyceraldehyde 3-phosphate dehydrogenase (GAPDH), which was used as a housekeeping gene control. Gene expression was examined by the 2^−ΔΔCt^ method [51].

### 4.14. Statistical Analysis

Each antiviral test was performed in triplicate and expressed as mean ± standard deviation (SD). One-way ANOVA was followed by the Dunnett’s multiple comparisons test, and all the graphs were generated by GraphPad Prism (version 8.0.1; software for 2D graphing and statistics; GraphPad Software Inc., San Diego, CA, USA, 2018). The significance of the difference between treated and CTR− samples was obtained with Dunnett’s test and ANOVA analysis. The *p*-value *< 0.05* was considered significant.

## 5. Conclusions

In this work, we successfully developed and characterized plant-mediated AgNPs embedded within GG/NaALG composite films, demonstrating their potential as innovative biomaterials for topical applications against HSV-1. The hydrogels exhibited excellent swelling behavior, water holding capacity, and thermal stability, key properties for wound dressings designed to manage exudate and support tissue healing in HSV-1–associated skin lesions. Notably, the GG_1.6_-NaALG_0.4_/AgNPs formulation released the highest amount of Ag^+^ ions, a factor that correlated directly with its superior antiviral performance. Across all antiviral assays, AgNP-loaded films significantly inhibited HSV-1 infection, with the most potent effects observed during virus pre-treatment and attachment phases. These findings suggest a possible interaction of AgNPs with viral particles during the early stages of infection, potentially involving the viral envelope or surface glycoproteins, thereby reducing viral adhesion. However, no direct mechanistic evidence (e.g., binding or imaging studies) was provided in this work, and further investigations are required to confirm this hypothesis. The improved antiviral efficacy of the films compared to free AgNPs or UU–based formulations further highlights the synergistic contribution of the polymeric network, which enhances nanoparticle stability, availability, and bioactivity. Overall, the combination of favorable physicochemical properties, controlled silver release, and potent antiviral activity positions these AgNP-based hydrogels as promising candidates for topical and wound care new strategies. Future work should focus on exploring functional surface modifications and validating therapeutic potential in vivo models to advance their translational applicability.

## Figures and Tables

**Table 1:** IC_50_ values for each antiviral assay.

Antiviral Assay	GC_1.6_-NaALG_0.4_/AgNPs	GC_1.2_-NaALG_0.8_/AgNPs	AgNPs
Co-treatment assay	1.392 µg/mL	2.239 µg/mL	9.1 × 10^5^ N° particles/mL
Virus pre-treatment	0.7820 µg/mL	1.018 µg/mL	3.9 × 10^5^ N° particles/mL
Attachment assay	0.6116 µg/mL	0.6971 µg/mL	2.9 × 10^5^ N° particles/mL
Entry assay	17.84 µg/mL	19.50 µg/mL	1.5 × 10^6^ N° particles/mL
Selectivity index (SI)	>150	>57	>17

Note: IC_50_ values are expressed using different units depending on the sample type and the analytical method employed. For AgNPs in suspension, concentrations are reported as particles/mL, as determined by nanoparticle tracking analysis, which directly measures nanoparticle number. For AgNP-loaded hydrogel films, IC_50_ values are expressed in µg/mL and refer to the concentration of silver released after immersion in PBS, as quantified by physicochemical analysis (atomic absorption spectroscopy). Therefore, these units are not directly comparable, as they reflect distinct measurement approaches and experimental conditions. Selectivity index (SI) values are reported as lower bounds (SI > CC_50_/IC_50_), calculated using the highest non-cytotoxic concentration tested.

**Table 2:** Composition (expressed as weight percent of the total weight of the components of the film) of the prepared materials; ⁂ AgNPs were added at a concentration of 2 × 10^7^ nanoparticles/mL.

Film Code	Weight/Volume Percentage (% *_w_*_/*v*_)				
	GG	NaALG	Tartaric Acid	Glycerol	AgNPs
GG_1.6_-NaALG_0.4_	50.0	12.5	6.25	31.25	--
GG_1.6_-NaALG_0.4_/AgNPs	50.0	12.5	6.25	31.25	⁂
GG_1.2_-NaALG_0.8_	37.5	25.0	6.25	31.25	--
GG_1.2_-NaALG_0.8_/AgNPs	37.5	25.0	6.25	31.25	⁂

**Table 3:** Oligos sequences for viral genes.

Gene Symbol	Forward Sequence (5′ → 3′)	Reverse Sequence (5′ → 3′)
HSV-1 UL54	TGGCGGACATTAAGGACATTG	TGGCCGTCAACTCGCAG
HSV-1 UL52	GACCGACGGGTGCGTTATT	GAAGGAGTCGCCATTTAGCC
HSV-1 UL27	GCCTTCTTCGCCTTTCGC	GCCTTCTTCGCCTTTCGC
GAPDH	CCTTTCATTGAGCTCCAT	CGTACATGGGAGCGTC

## Data Availability

The data presented in this study are available upon request from the corresponding author. Authors can confirm that all relevant data are included in the article.

## Supplementary Materials

The following supporting information can be downloaded at https://www.mdpi.com/article/10.3390/molecules31091459/s1: Figure S1. Evaluation of the two UU films, referred to as GG1.6-NaALG0.4/uva ursi and GG1.2-NaALG0.8/uva ursi, and of UU cytotoxicity; Figure S2. Time-shift assays of the two AgNPs films (A,C), GG1.6-NaALG0.4/AgNPs and GG1.2-NaALG0.8/AgNPs, and of AgNPs (B,D) to evaluate their potential antiviral effect against HSV-1; Figure S3. Time-shift assay of the two UU films, referred to as GG1.6-NaALG0.4/uva ursi and GG1.2-NaALG0.8/uva ursi, and of UU to evaluate their potential antiviral effect against HSV-1; Figure S4. Temperature-shift assay of the two UU films, referred to as GG1.6-NaALG0.4/uva ursi and GG1.2-NaALG0.8/uva ursi, and of UU to evaluate their potential antiviral effect against HSV-1; Table S1. IC50 values for each antiviral assay of the two UU films, referred to as GG1.6-NaALG0.4/uva ursi and GG1.2-NaALG0.8/uva ursi, and of UU.

## Abbreviations

The following abbreviations are used in this manuscript:

Ag+	Silver ion
AgNPs	Silver nanoparticles
AgNO_3_	Silver nitrate
CMC	Carboxymethylcellulose
CTR−/CTR+	Negative control/positive control
cP	Centipoise
Ct	Cycle threshold
DMSO	Dimethyl sulfoxide
DSC	Differential scanning calorimetry
FBS	Fetal bovine serum
FTLA	Finite track length adjustment
GG	Gellan gum
HNO_3_	Nitric acid
HSV-1/HSV-2	Herpes simplex virus (type 1/2)
IC_50_	Inhibitory concentration 50%
Milli-Q^®^	Ultrapure water system
MOI	Multiplicity of infection
MTT	3-(4,5-dimethylthiazol-2-yl)-2,5-diphenyltetrazolium bromide
NaALG	Sodium alginate
NaH_2_PO_4_	Sodium dihydrogen phosphate
Na_2_HPO_4_	Disodium hydrogen phosphate
nm	Nanometers
NTA	Nanoparticle tracking analysis
PBS	Phosphate-buffered saline
pfu/mL	Plaque-forming units per milliliter
RT-PCR	Real-Time Polymerase Chain Reaction
rpm	Rotations per minute
SD	Standard deviation
SWF	Simulated wound fluid
TGA	Thermogravimetric analysis
TEM	Transmission electron microscopy
UU	*Arctostaphylos uva-ursi* leaf extract
WHC	Water holding capacity
WU%	Water uptake percentage
XRD	X-ray Diffraction
