# Supplementary material for: Gellan Gum/Alginate Films Containing Biogenic uva ursi Silver Nanoparticles: Analytical Characterization and Antiviral Activity Against HSV-1
Source: Molecules. 2026 Apr 28;31(9):1459. doi: 10.3390/molecules31091459 (PMC13164898; doi:10.3390/molecules31091459)
Supplement: Supplementary file 1 [file molecules-31-01459-s001.zip › molecules-4259941-supplementary.pdf]

molecules

Supplementary Materials

Gellan Gum/Alginate Films Containing Biogenic *uva ursi* Silver Nanoparticles: Analytical Characterization and Antiviral Activity Against HSV-1

Roberta Della Marca [1,†], Francesco Busto [2,3,†], Carla Zannella [1,4], Stefano Liotino [2,3], Maria Chiara Sportelli [2], Muhammad Shoaib [1], Shahab Bashir [1], Massimiliano Galdiero [1,4], Elvira De Giglio [2,3,*] and Anna De Filippis [1,4,*]

1 Department of Woman, Child and General and Specialized Surgery, University of Campania "Luigi Vanvitelli", 80138 Naples, Italy; roberta.dellamarca@unicampania.it (R.D.M.); carla.zannella@unicampania.it (C.Z.); muhammad.shoaib1@unicampania.it (M.S.); shahab.bashir@unicampania.it (S.B.); massimiliano.galdiero@unicampania.it (M.G.)
2 Department of Chemistry, University of Bari Aldo Moro, Via Orabona 4, 70126 Bari, Italy; francesco.busto@uniba.it (F.B.); stefano.liotino@uniba.it (S.L.); maria.sportelli@uniba.it (M.C.S.)
3 INSTM, National Consortium of Materials Science and Technology, Via G. Giusti 9, 50121 Florence, Italy
4 UOC Virology and Microbiology, University Hospital "Luigi Vanvitelli", 80138 Naples, Italy
* Correspondence: elvira.degiglio@uniba.it (E.D.G.); anna.defilippis@unicampania.it (A.D.F.)
† These authors contributed equally to this work.

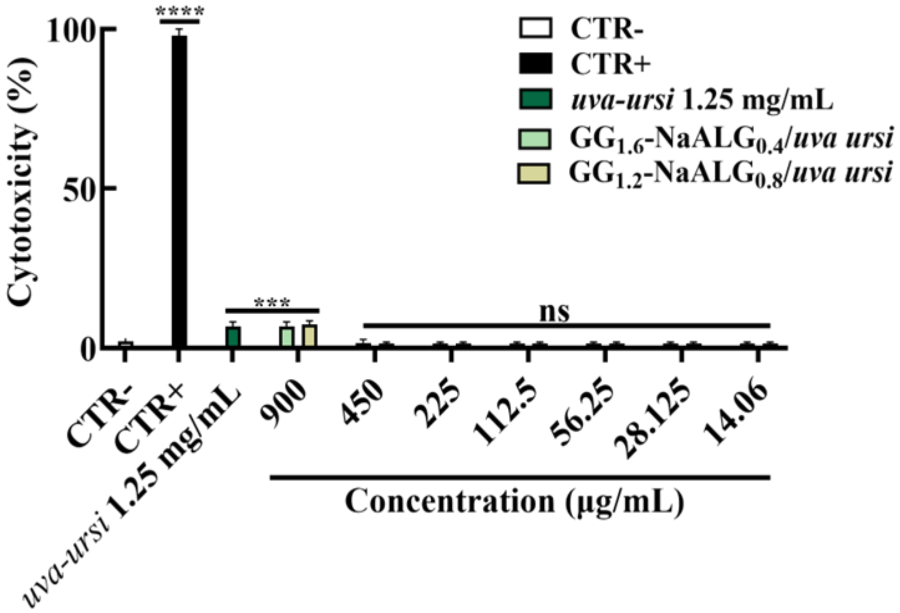

Figure S1. Evaluation of the two UU films, referred to as $GG_{1.6}$-$NaALG_{0.4}$/*uva ursi* and $GG_{1.2}$-$NaALG_{0.8}$/*uva ursi*, and of UU cytotoxicity. Cytotoxicity was assessed in Vero 76 cells using an MTT assay after 24 h of treatment with the sample suspension. UU was tested at 1.25 mg/mL. The two films $GG_{1.6}$-$NaALG_{0.4}$ and $GG_{1.2}$-$NaALG_{0.8}$ were overloaded with 0.9 mg of UU, immersed in 1 mL of PBS for 24h, and tested the day after in 2-fold dilutions. The data represent the mean ± SD. CTR −: untreated cells; CTR +: 100% DMSO. Statistical analysis was performed using Dunnett's multiple comparisons test: **** p-value < 0.0001; *** p-value= 0.0004; ns p-value > 0.05.

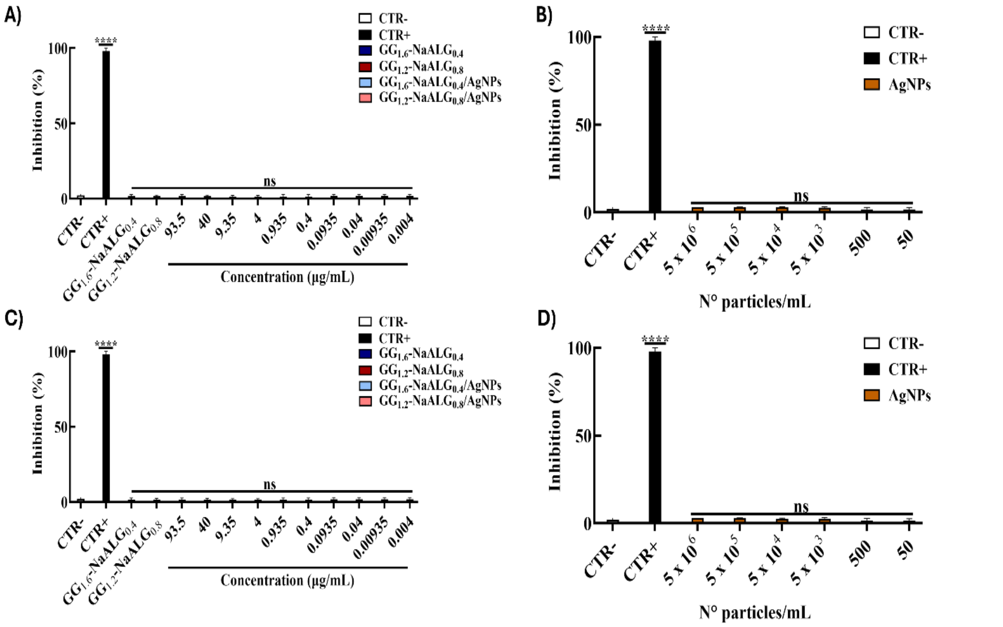

Figure S2. Time-Shifts assays of the two AgNPs films (A, C), GG$_{1.6}$-NaALG$_{0.4}$/AgNPs and GG$_{1.2}$-NaALG$_{0.8}$/AgNPs, and of AgNPs (B, D) to evaluate their potential antiviral effect against HSV-1. Two different plaque reduction assays are shown. **(A, B)** Cell pre-treatment assay; **(C, D)** Post-treatment assay. The untreated cells represented the negative control (CTR−). Dextran-sulfate (1 μM) in cell pre-treatment, and aciclovir (5 μM) in post-treatment were used as positive control (CTR+). Data represent mean ± standard deviation (SD) of three independent experiments. **** *p*-value < 0.0001; ns p-value > 0.05.

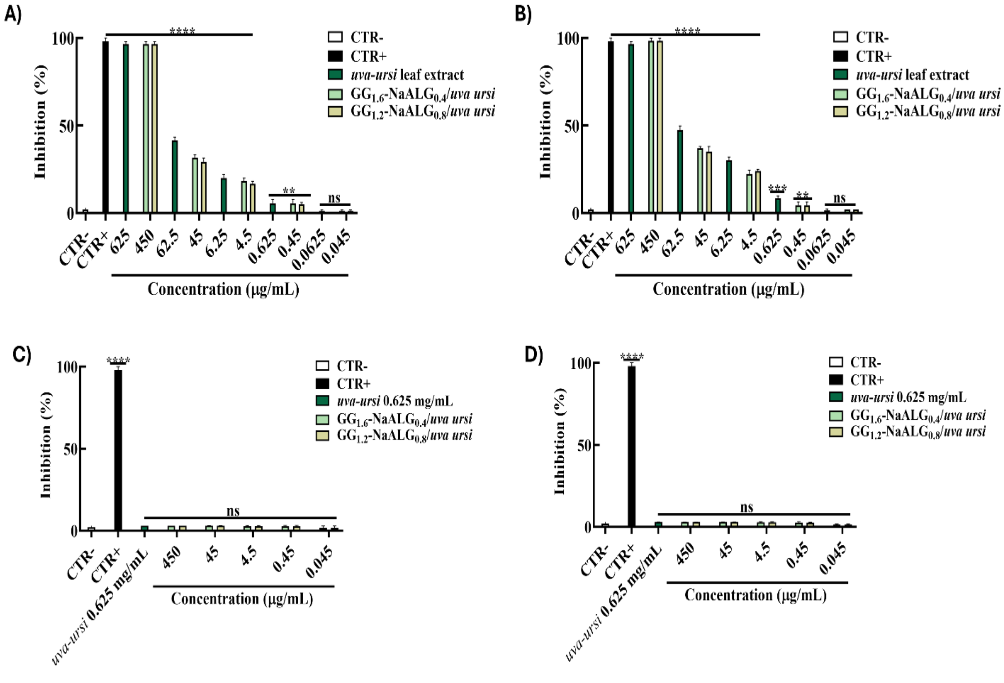

Figure S3. Time-Shifts assay of the two UU films, referred to as GG$_{1.6}$-NaALG$_{0.4}$/*uva ursi* and GG$_{1.2}$-NaALG$_{0.8}$/*uva ursi*, and of UU to evaluate their potential antiviral effect against HSV-1. Four plaque reduction assays are shown. **(A)** Co-treatment assay; **(B)** Virus pre-treatment assay; **(C)** Cell pre-treatment assay; and **(D)** Post-infection assay. UU was tested at 0.625 mg/mL in 2-fold serial dilutions. The two films GG$_{1.6}$-NaALG$_{0.4}$ and GG$_{1.2}$-NaALG$_{0.8}$ were overloaded with 0.9 mg of UU, immersed in 1 mL of PBS for 24h, and tested the day after from 450 μg/mL in 2-fold dilutions.

The untreated cells represented the negative control (CTR−). Several compounds were used as positive controls (CTR+): melittin (5 μM) in the co-treatment and virus pre-treatment, dextran-sulfate (1 μM) in cell pre-treatment, and acyclovir (5 μM) in post-treatment. Data represent mean ± standard deviation (SD) of three independent experiments. **** *p*-value < 0.0001; *** *p*-value= 0.0004; ** *p*-value=0.0042; ns p-value > 0.05.

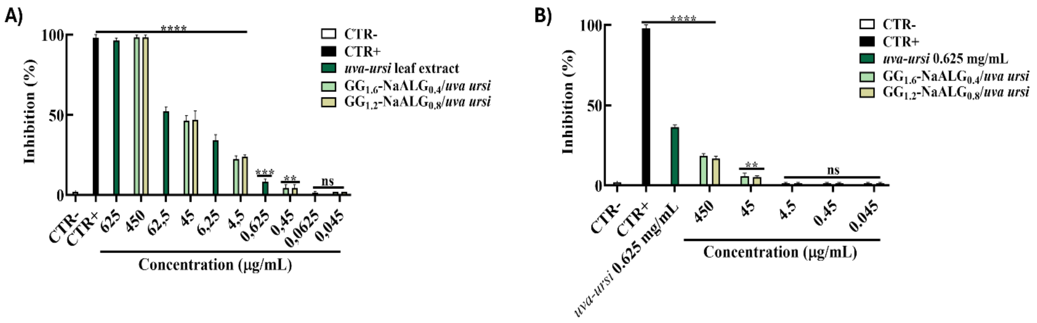

Figure S4. Temperature-Shifts assay of the two UU films, referred to as $GG_{1.6}$-$NaALG_{0.4}$/*uva ursi* and $GG_{1.2}$-$NaALG_{0.8}$/*uva ursi*, and of UU to evaluate their potential antiviral effect against HSV-1. Two different plaque reduction assays are shown. **(A)** Attachment assay; **(B)** Entry assay. Untreated cells served as the negative control (CTR−), while heparin (1 mg/mL) was used as the positive control (CTR+) in both assays. Uva ursi was tested at 0.625 mg/mL in 2-fold serial dilutions. The two films $GG_{1.6}$-$NaALG_{0.4}$ and $GG_{1.2}$-$NaALG_{0.8}$ were overloaded with 0.9 mg of UU, immersed in 1 mL of PBS for 24h, and tested the day after from 450 μg/mL in 2-fold dilutions. Data represent mean ± standard deviation (SD) of three independent experiments. **** *p*-value < 0.0001; *** *p*-value= 0.0004; ** *p*-value=0.0042; ns p-value > 0.05.

Table S1. IC_{50} values for each antiviral assay of the two UU films, referred to as $GG_{1.6}$-$NaALG_{0.4}$/*uva ursi* and $GG_{1.2}$-$NaALG_{0.8}$/*uva ursi*, and of UU.

Antiviral assay	Uva ursi leaf extract (UU)	$GG_{1.6}$-$NaALG_{0.4}$/*uva ursi*	$GG_{1.2}$-$NaALG_{0.8}$/*uva ursi*
Co-treatment assay	59.63 μg/mL	72.07 μg/mL	76.98 μg/mL
Virus pre-treatment	38.38 μg/mL	52.72 μg/mL	54.75 μg/mL
Attachment assay	28.73 μg/mL	34.06 μg/mL	36.56 μg/mL
Entry assay	-	-	-
